# Evaluation of the correlation of vasculogenic mimicry, ALDH1, KAI1 and microvessel density in the prediction of metastasis and prognosis in colorectal carcinoma

**DOI:** 10.1186/s12893-017-0246-6

**Published:** 2017-04-21

**Authors:** Bo Zhu, Lei Zhou, Lan Yu, Shiwu Wu, Wenqing Song, Xiaomeng Gong, Danna Wang

**Affiliations:** Department of Pathology, the First Affiliated Hospital of Bengbu Medical College, Bengbu Medical College, No.287, Changhuai Road, Bengbu, Anhui Province China

**Keywords:** CRC, VM, ALDH1, KAI1, MVD, Prognosis

## Abstract

**Background:**

Metastasis and recurrence are the most common reasons for treatment failure of colorectal carcinoma (CRC). Vasculogenic mimicry (VM, blood supply formation often seen in highly aggressive tumors), Aldehyde dehydrogenase 1 (ALDH1, a biomarker of cancer stem cells), KAI1 (a suppressor gene of tumor metastasis) are all valuable factors for metastasis and prognosis in diverse human cancers. However, the correlation of VM, ALDH1, KAI1 and microvessel density (MVD) in CRC is unclear. In this study, we analyzed the correlations among VM, ALDH1, KAI1 and MVD, as well as their respective correlations with clinicopathological parameters and survival in CRC.

**Methods:**

The level of VM, ALDH1, KAI1 and MVD in 204 whole tissue samples of CRC were examined by immunhistochemistry. Clinical data was also collected.

**Results:**

Levels of VM, ALDH1 and MVD were significantly higher, and levels of KAI1 significantly lower, in CRC tissues than in normal colorectal tissues. Levels of VM, ALDH1 and MVD were positively associated with invasion of depth, lymph node metastasis (LNM), distant metastasis and tumor-node-metastasis (TNM) stages, and negatively with patients’ overall survival (OS). Levels of KAI1 was negatively correlated with invasion of depth, LNM, distant metastasis and TNM stages, and the KAI1 positive expression subgroup had significantly longer OS than did the KAI1- subgroup. In multivariate analysis, high levels of VM, ALDH1 and KAI1, as well as TNM stages were independently correlated with lower OS in patients with CRC.

**Conclusions:**

VM, MVD and the expression of ALDH1 and KAI1 may represent promising metastatic and prognostic biomarkers, as well as potential therapeutic targets for CRC.

## Background

In 2012, colorectal cancer (CRC) was reportedly found in about 1.4 million and accounted for approximately 10% of all new cancer cases, making it the third most commonly diagnosed cancer in the worldwide [1]. The increase in China may reflect an increased prevalence of risk factors for CRC, such as unhealthy diet, obesity and smoking [2, 3]. In China, majority of patients diagnosed with CRC have advance stage cancer and are unsuitable for curative therapy.

The most common reasons for cancer treatment failure are metastasis and recurrence. This may be related to a small population of tumor cells which named as cancer stem cells (CSC) or tumor initiating cells (TIC). CSC has the capacity to self-renew and give rise to progression and differentiation in various human solid tumors [4–7]. CSC has been isolated from various tumor entities and related to therapeutic (chemo- or radio-resistance) resistance and poor prognosis. Aldehyde dehydrogenases (ALDHs), also known as a family enzymes, which can be found in the mitochondria, nucleus and cytoplasm [8]. The ALDH enzymes can modulate several fundamental biological functions, such as proliferation and differentiation, as well as the cell response to oxidative stress. ALDH1, which is an important member of ALDH family enzymes, is considered as a marker for CSC. The function of ALDH1 is to detoxify and metabolize various endogenous and exogenous aldehydes, as well as oxidize retinol to synthesize retinoic acid [9]. Overexpression of ALDH1 may increase the risk of alcohol-linked cancers [10]. Furthermore, ALDH1 has been considered as a useful marker for metastasis and poor prognosis in various malignant tumors, including pancreatic cancer, esophageal cancer, lung cancer and gastric cancer [11–14].

Angiogenesis may also be related to metastasis and recurrence. Microvessel density (MVD) is one of the most commonly indicators for assessing the activity of angiogenesis. However, the role of MVD that predicts prognosis was controversial in some studies [15, 16]. Similar to the role, the clinical benefit of anti-angiogenic therapy for malignant tumors is still unsatisfactory [17]. It is critical to be addressed whether there were other mechanisms about tumor blood supply. In 1999, Maniotis et al. firstly reported vasculogenic mimicry (VM) [18], a new blood supply is a vascular channel-like structure that is lining of cancer cells. VM is consisted of three structures: highly aggressive cancer cells, rich extracellular matrix and vasculogenic-like channel to the host microcirculation system [19–22]. VM, as a supplementary theory of angiogenesis, may explain the failure of anti-angiogenic therapy [23, 24]. Recently, accumulating evidence has been suggested that VM should be considered as a valuable biomarker for metastasis and prognosis in various human cancers [25–29].

KAI1, also named as CD82, which is originally considered as a suppressor gene of metastasis in prostate cancer cells [30]. KAI1, which belongs to the tetraspanin superfamily (TM4SF), is located on chromosome 11p11.2 and contains 10 exons and 9 introns. It has been demonstrated that TM4SF protein could inhibit tumor metastasis [31]. KAI1 can inhibit tumor metastasis through promoting cell-cell or cell-extracellular matrix interactions [32]. KAI1 is also involved in some fundamental biological processes, including migration, adhesion, differentiation and invasion [33, 34]. KAI1 is also identified as a useful biomarker for metastasis and prognosis in diverse human cancers [35].

Overall, studies of ALDH1, VM, MVD and KAI1 in association to metastasis and prognosis suggested that these biomarkers should affect tumor progression. However, correlations among ALDH1, VM, MVD and KAI1 in CRC have not yet been widely reported. In this study, we verified the hypothesis that above biomarkers are mutual related and associated with metastasis and prognosis in CRC.

## Methods

### Patients and tissue samples

We collected samples from all 204 patients (median age: 59.4 years; range: 31–77 years) who were treated for CRC at the Department of Pathology of the First Affiliated Hospital of Bengbu Medical College, from January 2007 to December 2010, along with 204 samples of the corresponding adjacent normal colorectal mucosa tissues (removed the same patient, from surrounding colorectal mucosa tissue at least 5 cm away from the cancer edge). Patients who had received preoperative chemo- or radio-therapy were excluded. All tissue specimens were obtained with patients writing consent and the study was approved by the ethical committee of the Bengbu Medical College and performed in accordance with the guidelines of the Declaration of Helsinki. We collected the entirely clinicopathological and follow-up data (at 6 months intervals by mail, phone or social application). Overall survival (OS) time was counted from the patients operation date to his/her death date or January 2015 (mean OS: 51.6 months; range: 8–96 months). Grade of tumor differentiation was according to WHO (World Health Organization) standard. Tumor-node –metastasis stage was assessed according to the 7^th^ edition of the AJCC (American Joint Committee on Cancer). Other characteristics see Table 1.

**Table 1:** Patients characteristics

Patients characteristics	Frequency (*n*)	Percentage (%)
Gender		
Male	120	58.8
Female	84	41.2
Ages		
< 60	81	39.7
≥ 60	123	60.3
Size		
< 5.0 cm	119	58.3
≥ 5.0 cm	85	41.7
Location		
Colon	99	48.5
Rectum	105	51.5
Grade		
Well	45	22.1
Moderate	107	52.5
Poor	52	25.5
Invasion		
Subserosa	113	55.4
Visceral peritoneum	91	44.6
Distant metastasis		
No	176	86.3
Yes	28	13.7
Lymph node metastasis		
No	122	59.8
Yes	82	40.2
TNM stage		
Iand II	125	61.3
III and IV	79	38.7

### Immunohistochemistry

Immunohistochemistry was conducted according to the guideline of Elivision™ Plus detection kit instructions (Lab Vision, USA). All CRC- and corresponding normal colorectal mucosa tissues were fixed in 10% buffered formalin and embedded in paraffin. Then continuous 4 μm thick tissue sections were cut. All specimens were deparaffinized and dehydrated with xylene and graded alcohol, subsequently washed for 10 min with PBS (phosphate buffer solution, pH 7.2). Endogenous peroxidase activity was quenched by incubation of samples in methanol containing 3% H_2_O_2_ for 10 min at room temperature (RT), then placed in citrate buffer (pH 6.0) and heated to 95 °C for 30 min for antigen repair. After several washes with PBS, all samples were blocked with goat serum for 20 min at RT, then incubated with mouse monoclonal antibody against human ALDH1 (Abcam, USA) or CD34 (Abcam, USA) or KAI1 (Abcam, USA) for 1 h at 37 °C. Microvessel density (MVD) was determined by the number of small CD34 positive vessels counted. All sections were performed periodic acid-Schiff (PAS) -CD34 dual staining to characterize endothelial cells in glycosylated basement membranes of vessels, as well as vasculogenic-like structures [19]. Furthermore, there was no necrosis or hemorrhage near the VM channels in cancer tissues. All samples were counterstained with hematoxylin, dehydrated, air-dried and mounted. The method was adopted from Weidner et al. with some modifications to assess the MVD of CRC [36]. A modified Yue’s method was used to evaluate the VM of CRC [37]. ALDH1 positive staining was mainly confined in the cytoplasm of cancer cells; KAI1 positive staining was mainly confined in the membrane and cytoplasm of cancer cell. Negative controls were prepared by leaving out primary antibodies from the staining procedure.

### Evaluation of staining

Immunotaining findings were explained semi-quantitatively by two independent pathologists who were blind to the clinical, pathological and follow-up data. To avoid the intratumoral heterogeneity of antibodies expression, ten representative areas at high-power-fields (HPF) from different areas of each CRC’s section were detected. immunohistochemistry results were counted according to the extent and intensity. The immunostaining intensity scores were graded as follows: 0, none; 1, weak; 2, moderate; and 3, strong. The immunostaining extent scores were graded as follows: 1, <11%; 2, 11–50%; 3, 51–75%; and 4, >75%. Then, the intensity and extent scores were multiplied to reach a final score that ranged from 0 to 12. The scores ≥3 was considered positive. For samples that were positive for both ALDH1 and KAI1, an average of the final score of each slide was taken.

### Statistical analysis

Correlations between clinicopathological variables and ALDH1, VM, MVD or KAI1 were compared using Fisher’s exact test or Chi-square test. The correlations among ALDH1, or VM, or MVD or KAI1 were compared using Spearman’s coefficient test. The effects of ALDH1, VM, MVD or KAI1 on survival were determined using univariate and multivariate analyses. Independent prognostic factors were determined by the Cox regression model for multivariate analysis. The Kaplan-Meier method with log-rank test for univariate overall survival analysis was used to assess the correlation between ALDH1+, VM+, MVD+ or KAI1+ and clinicopathological variables using SPSS 19.0 software for Windows (Chicago, IL). A value of *P* < 0.05 was defined as statistically significant.

## Results

### Correlations between ALDH1, VM, MVD or KAI1 and clinicopathological variables

**Fig. 1:**
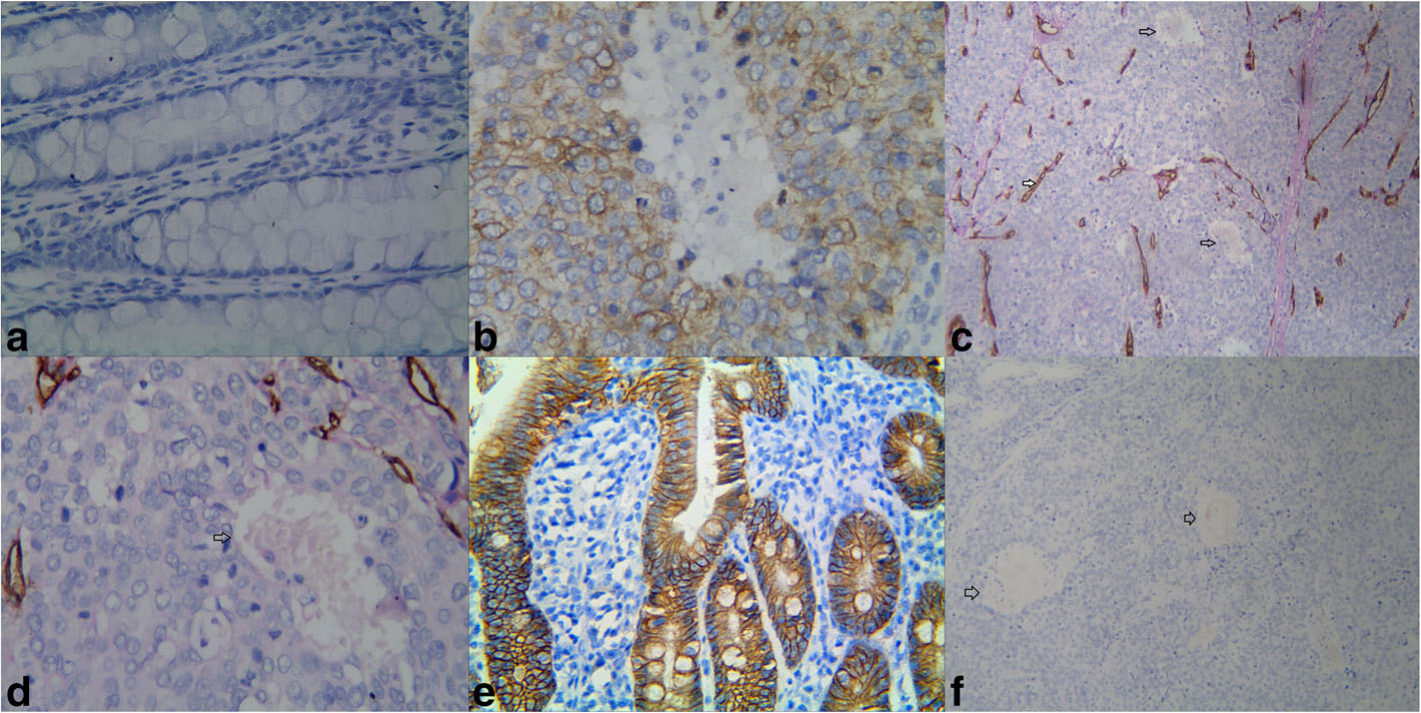
Immunostaining of ALDH1, or VM or KAI1 in CRC or the control tissue. **a** Negative staining ALDH1 in the control tissue (400 magnification); **b** Positive staining of ALDH1 in the cytoplasm of cancer cells (400 magnification); **c** Positive staining of VM in the colorectal carcinoma tissues (100 magnification, *white arrow* is VM structure, *black arrow* is microvessel); **d** Positive staining of VM in the colorectal carcinoma tissues (400 magnification, *white arrow* is VM structure); **e** Positive staining of KAI1 in the membrane of control tissues (400 magnification); **f** Negative staining of KAI1 in the colorectal carcinoma tissues (100 magnification, Fig. **b**, **c**, **d**, **f** are serial sections)

To assess the contributions of ALDH1, VM, MVD and KAI1 to CRC, the results thereof were immunohistochemically assessed for both CRC and normal colorectal mucosa tissue samples. These data were then compared to the clinicopathological variables. The positive rate of ALDH1 expression in the CRC samples (73.5%, 150/204) was significantly higher than that in the control normal tissues (6.9%, 14/204; *P* < 0.001; Fig. 1a and b). The positive expression rate of ALDH1 in CRC was positively correlated with tumor invasion, lymph node metastasis, distant metastasis and TNM stage, but not with patients age, gender, tumor size, grade or location (Table 2).

**Table 2:** The correlation between ALDH1, or VM, or MVD or KAI1 and clinicopathological characteristics in colorectal carcinoma

Variable	ALDH1		*P*	VM		*P*	MVD		*P*	KAI1		*P*
	Negative	Positive		Negative	Positive		Mean F			Negative	Positive	
Gender			0.805			0.742		0.539	0.464			0.590
Male	31	89		77	43		21.4 ± 5.8			80	40	
Female	23	61		52	32		20.8 ± 5.7			59	25	
Age (years)			0.150			0.948		1.074	0.301			0.579
< 60	17	64		51	30		21.7 ± 6.3			57	24	
≥ 60	37	86		78	45		20.8 ± 5.4			82	41	
Size (cm)			0.629			0.508		0.062	0.804			0.780
< 5.0	30	89		73	46		21.1 ± 6.2			82	37	
≥ 5.0	24	61		56	29		21.3 ± 5.2			57	28	
Location			0.066			0.324		0.606	0.437			0.891
Rectum	22	83		63	42		21.5 ± 5.7			72	33	
Colon	32	67		66	33		20.8 ± 5.9			67	32	
Grade			0.555			0.364		2.903	0.057			0.001
Well	14	31		28	17		20.3 ± 4.5			20	25	
Moderate	25	82		72	35		22.1 ± 5.8			80	27	
Poor	15	37		29	23		20.0 ± 6.4			39	13	
Invasion			0.024			0.002		11.858	0.001			0.016
Subseroa	37	76		82	31		19.9 ± 4.7			69	44	
Visceral peritoneum	17	74		47	44		22.7 ± 6.6			70	21	
Distant metastasis			0.006			<0.001		34.878	<0.001			0.018
No	53	123		121	55		2.3 ± 5.1			114	62	
Yes	1	27		8	20		26.7 ± 6.7			25	3	
LNM			0.002			<0.001		14.053	<0.001			<0.001
No	42	80		92	30		20.0 ± 4.9			70	52	
Yes	12	70		37	45		22.9 ± 6.4			69	13	
TNM stage			0.024			<0.001		34.116	<0.001			<0.001
Iand II	40	85		100	25		19.4 ± 4.3			67	58	
III and IV	14	65		29	50		23.9 ± 6.7			72	7	

Similar to ALDH1, the positive rate of VM (Small vessel-like lumen in CRC that were PAS-positive but CD34-negative were to be VM. The VM channels pattern included linear, tubular, and network and so on.) was significantly higher in CRC (36.8%, 75/204) than that in the control tissues (0%, 0/204; *P* < 0.001, Fig. 1c and d). The positive rate of VM in CRC was positively correlated with tumor invasion, LNM, distant metastasis and TNM stage, but not patients age, gender, tumor size, grade or location (Table 2). And the positive staining of MVD scores were found to be significantly correlated with tumor invasion, LNM, distant metastasis and TNM stage in CRC. However, the scores of MVD were no significant association with patient age, gender, tumor size, tumor grade and location (Table 2).

The positive rate of KAI1 expression was significantly lower in CRC tissues (31.9%, 65/204) than that in control normal tissues (98.0%, 200/204; *P* < 0.001, Fig. 1e and f). The positive rate of KAI1 expression was inversely correlated with tumor grade, invasion, LNM, distant metastasis and TNM stage. No correlation was found between KAI1 expression and patients age, gender, tumor size or location (Table 2).

### Univariate and multivariate analysis

**Fig. 2:**
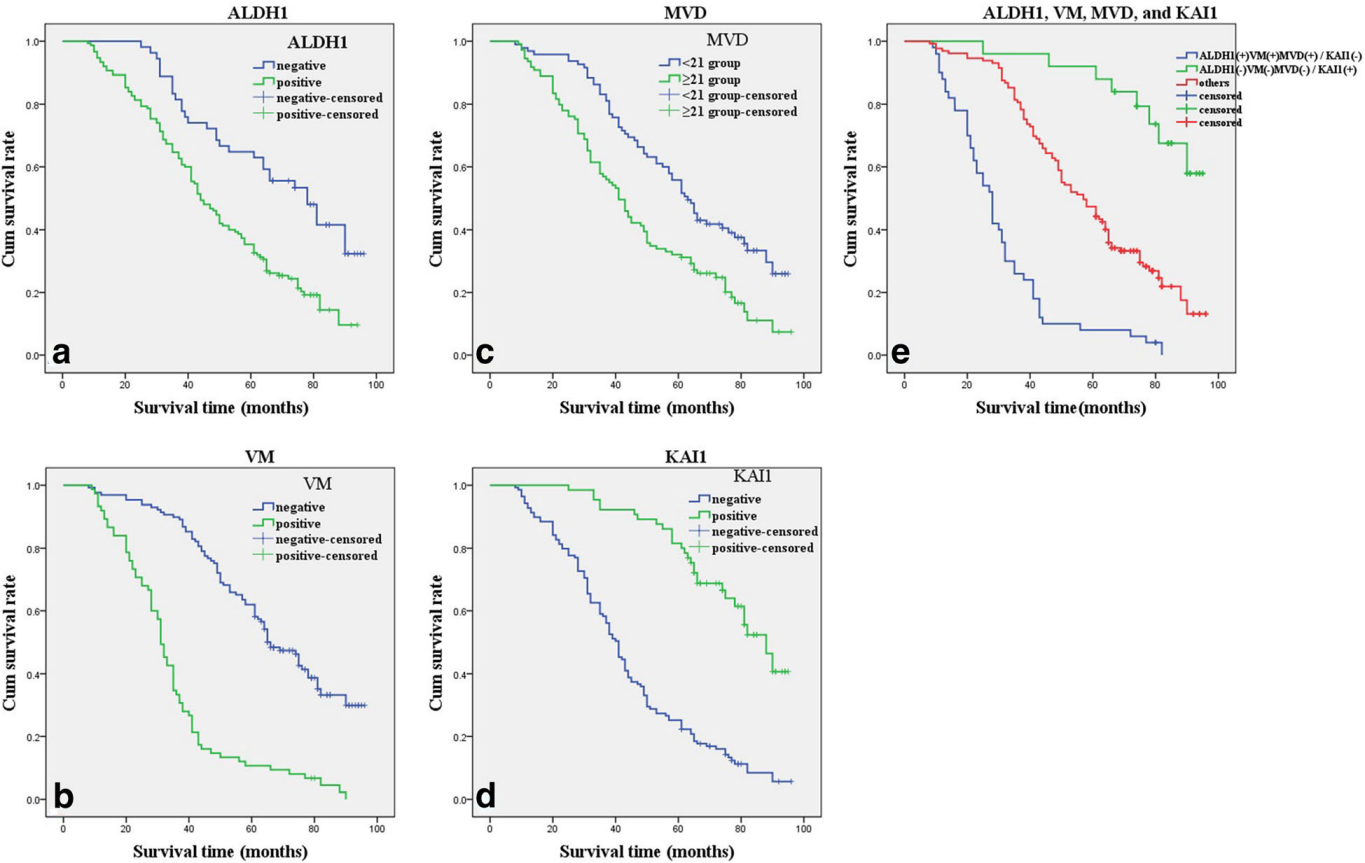
Kaplan-Meier analysis of the survival rate of patients with colorectal carcinoma. **a** Overall survival of all patients in relation to ALDH1 expression (log-rank = 16.908, *P* < 0.001); **b** Overall survival of all patients in relation to VM (log-rank = 86.416, *P* < 0.001); **c** Overall survival of all patients in relation to MVD (log-rank = 15.610, *P* < 0.001); **d** Overall survival of all patients in relation to KAI1 expression (log-rank = 60.613, *P* < 0.001). In **a**, **b**, **c** and **d** analyses, the *green line* represents positive staining of factors (MVD score ≥21 is positive) and the *blue line* represents negative staining factors (MVD score <21 is negative). **e** Overall survival of all patients in relation to the combination of KAI1, ALDH1, VM and MVD (log-rank = 97.184, *P* < 0.001). The *green line* represents positive expression of KAI1 and negative expression of ALDH1, VM and MVD and the *blue line* represents negative expression of KAI1 and positive expression of ALDH1, VM and MVD. The *red line* represents other positive or negative expression of the proteins

Follow-up data showed that OS time was significantly shorter in CRC patients with positive expression of ALDH1 (47.1 ± 22.4 months) compared with those with ALDH1-negative (64.3 ± 21.9 months; log-rank = 16.908, *P* < 0.001; Fig. 2a). Similarly, the OS time of VM-positive patients (34.7 ± 19.0 months) was significantly lower than those of VM-negative patients (61.5 ± 20.0 months; log-rank = 86.416, *P* < 0.001; Fig. 2b). The OS time of MVD-positive (the mean score of MVD is 21.2 ± 5.8, so MVD score ≥21 is considered positive, MVD score <21 is considered negative) patients (44.9 ± 22.8 months) was significantly shorter than those who were MVD-negative group (59.4 ± 21.9 months; log-rank = 15.610, *P* < 0.001; Fig. 2c). The OS time of KAI1-positive patients (70.4 ± 16.1 months) was significantly longer than those who were KAI1-negative (42.9 ± 21.2 months; log-rank = 60.613, *P* < 0.001; Fig. 2d). The combination of KAI1 negative expression and positive expression of ALDH1, VM and MVD had a poorer prognosis than did the reverse combination (log-rank = 97.184, *P* < 0.001; Fig. 2e). In the univariate analysis, OS time was significantly correlated with clinicopathological variables, including invasion (*P* = 0.002, log-rank = 9.604), LNM (*P* < 0.001, log-rank = 19.908), and TNM stage (*P* < 0.001, log-rank = 53.120) (Table 3)

**Table 3:** Results of univariate analyses of overall survival (OS) time

Variable	*n*	Mean OS (months)	Log-rank	*P* value
ALDH1			16.908	<0.001
Negative	54	64.3 ± 21.9		
Positive	150	47.1 ± 22.4		
VM			86.416	<0.001
Negative	129	61.5 ± 20.0		
Positive	75	34.7 ± 19.0		
MVD			15.610	<0.001
< 21 group	95	59.4 ± 21.9		
≥ 21 group	109	44.9 ± 22.8		
KAI1			60.613	<0.001
Negative	139	42.9 ± 21.2		
Positive	65	70.4 ± 16.1		
Gender			0.174	0.677
Male	120	53.5 ± 21.9		
Female	84	49.0 ± 25.5		
Ages (year)			0.063	0.802
< 60	81	51.9 ± 23.3		
≥ 60	123	51.5 ± 23.7		
Size (cm)			0.392	0.531
< 5.0 ≥ 5.0	119 85	51.0 ± 23.3 52.5 ± 23.8		
Location			2.610	0.106
Rectum	105	49.7 ± 24.5		
Colon	99	53.7 ± 22.3		
Grade			1.266	0.531
Well	45	55.4 ± 24.7		
Moderate	107	51.4 ± 22.9		
Poor	52	48.9 ± 23.5		
Invasion			9.604	0.002
Subserosa	113	56.9 ± 22.6		
Visceral peritoneum	91	45.1 ± 22.9		
Distant metastasis			3.717	0.054
No	176	53.4 ± 23.2		
Yes	28	40.8 ± 22.7		
LNM			19.908	<0.001
No	122	59.4 ± 19.7		
Yes	82	40.0 ± 23.9		
TNM stage			53.120	<0.001
Iand II	125	60.3 ± 19.9		
III and IV	79	37.9 ± 22.2		

Multivariate analysis suggested that ALDH1 and KAI1 positive expression, VM, invasion, as well as TNM stage, were independent prognostic indicators for CRC (Table 4).

**Table 4:** Results of multivariate analyses of overall survival (OS) time

Variable	B	SE	P	RR	95% CI
Invasion	0.362	0.180	0.044	1.436	1.010–2.042
TNM stage	0.592	0.204	0.004	1.808	1.212–2.697
ALDH1	0.587	0.225	0.009	1.798	1.157–2.794
VM	0.912	0.206	<0.001	2.490	1.664–3.725
KAI1	−1.196	0.253	<0.001	0.302	0.184–0.497

### Association among ALDH1, VM, MVD and KAI1 in CRC

Spearman correlation coefficient analysis indicated a negative association between the positive expression of KAI1 and that of ALDH1 (*r* = −0.305, *P* < 0.001), or VM (*r* = −0.369, *P* < 0.001), or MVD (*r* = −0.458, *P* < 0.001). Expression of ALDH1 and that of VM (*r* = 0.181, *P* = 0.010), and MVD scores (*r* = 0.242, *P* < 0.001) were positively correlated, as were VM and MVD scores (*r* = 0.386, *P* < 0.001; Table 5).

**Table 5:** Correlation among ALDH1, VM, MVD and KAI1 in CRC

Variable	ALDH1		*r*	*P*	VM		*r*	*P*	MVD		*r*	*P*
	Negative	Positive			Negative	Positive			<21 group	≥21 group		
ALDH1							0.181	0.010			0.242	<0.001
Negative					42	12			36	18		
Positive					87	63			59	91		
VM			0.181	0.010							0.386	<0.001
Negative	42	87							79	50		
Positive	12	63							16	59		
KAI1			−0.305	<0.001			−0.369	<0.001			−0.458	<0.001
Negative	24	115			71	68			43	96		
Positive	30	35			58	7			52	13		

## Discussion

CRC is a highly heterogeneous tumor. This heterogeneity may influence the reproducibility of biomarker assessment [38, 39]. So, prognostic role of candidate biomarkers should be thoroughly assessed to guarantee their validity. ALDH1, an enzyme related to vitamin A metabolism, is a CSC biomarker in various cancers [11–14]. In this study, We found that ALDH1 expression was significantly correlated with tumor invasion, LNM, distant metastasis and TNM stage. Furthermore, Kaplan-Meier survival analysis showed that ALDH1-positive CRC patients had significantly shorter OS than did ALDH1-negative patients. Our findings were consistent with previous studies in CRC [11, 40, 41] suggesting that ALDH1 should be considered as a valuable biomarker for CRC.

VM should be involved in the process of progression and metastasis of various cancers [16, 19, 24–29], suggesting that VM should be considered as a potential candidate therapeutic target. In this study, We found that VM and MVD were positively related to tumor invasion, LNM, distant metastasis and TNM stage. Moreover, Kaplan-Meier survival analysis indicated that VM-positive or MVD-positive CRC patients had significantly shorter OS than did VM-negative or MVD-negative. These findings suggested that VM or MVD should be a useful biomarker for predicting progression and metastasis in CRC. Similar results are obtained from some other immunohistochemical studies which detected the metastatic and prognostic significance of VM in CRC patients [42–44].

KAI1 is extensively considered as a suppressor gene of tumor metastasis in various human cancers [30–35]. KAI1 can inhibit cell migration, differentiation, invasion and metastasis. In this study, we found that KAI1 expression was significantly lower in CRC tissues than that in the control tissues. And its positive expression as inversely associated with tumor grade, invasion, LNM, distant metastasis and TNM stage. Furthermore, Kaplan-Meier survival analysis showed that CRC patients with KAI1-positive expression had significantly longer survival time than did KAI1-negative patients. These findings suggested that down- or-lost regulation of KAI1 should promote CRC progression and metastasis, which are consistent with the previous studies [30–35, 45].

TNM stage provides therapeutic strategies for CRC patients, but not provides entirely information about CRC behavior. Therefore, it is urgent to find novel and effective biomarkers for predicting CRC patients progression, metastasis and prognosis. In this study, multivariate Cox model analysis showed that ALDH1+, KAI1+, VM+ and tumor invasion, as well as TNM stage, are independent prognostic factors for CRC patients. The most common causes of cancer-related deaths in CRC are metastasis and recurrence. Our findings thus demonstrated that ALDH1, VM and KAI1 should be considered as reliable biomarker for CRC, especially in predicting progression, metastasis and prognosis.

Furthermore, ALDH1 is a biomarker of CSC which should be involved in the initiation and progression of CRC. The niche where CSC reside is mainly composed of microvessles and microlymphatic vessels. Abnormal expression of ALDH1 may be involved in the initiation, development, invasion, metastasis of cancers [45, 46]. Some researchers found that CSC were capable of differentiation along tumor and endothelial cells [47, 48]. These findings demonstrated that these cells (tumor and endothelial cells) were derived from CSC, thus CSC also mimicked endothelial cells to form a vasculogenic-like network to convey nutrient and oxygen. In the same time, CSC were capable of differentiation along endothelial cells and stimulated angiogenesis in order to tumor growth and invasion. KAI1 could inhibit the process of epithelial-mesenchymal transition (EMT) to prevent angiogenesis [49]. KAI1 is a cell membrane protein that bind to ECM or adhesion. Thus, decreased expression of KAI1 lost its role of inhibiting tumor metastasis and angiogenesis. Overall, these findings suggested that there should be a complex association between ALDH1, VM, MVD and KAI1 in tumor progression and metastasis. Combined with the findings of this study, to some extent, we believed that the interaction of these biomarkers could reflect the biological behavior of CRC cells, thus providing a choice of therapeutic strategies target.

## Conclusions

It is suggested that ALDH1 should play a critical role in the evolution of CRC. The combined detection of ALDH1, VM, MVD and KAI1 should be valuable as biomarkers for metastasis and thereby prognosis for CRC patients.

## Abbreviations

AJCC: American Joint Committee on Cancer
ALDH1: Aldehyde dehydrogenase 1
CI: Confidence intervals
CRC: Colorectal carcinoma
CSC: Cancer stem cells
EMT: Epithelial-mesenchymal transition
HR: Hazard ration
LNM: Lymph node metastasis
MVD: Microvessel density
OS: Overall survival
PAS: Periodic acid-Schiff
PBS: Phosphate buffered saline
RT: Room temperature
TNM: Tumor node metastasis
VM: Vasculogenic mimicry
WHO: World Health Organization
